# Physically validated mitral valve models for surgical simulation: Bridging anatomy, pathology, and practice

**DOI:** 10.1016/j.xjon.2026.101746

**Published:** 2026-04-01

**Authors:** Patrick Carnahan, Charles Yuan, John Moore, Luca Zanella, Terry Peters, Elvis Chen, Gianluigi Bisleri

**Affiliations:** a Imaging Laboratories, Robarts Research Institute, Western University, London, Ontario, Canada; b Heart Surgery, Department of Medical and Surgical Sciences, University of Bologna, Bologna, Italy; c Department of Medical Biophysics, Western University, London, Ontario, Canada; d School of Biomedical Engineering, Western University, London, Ontario, Canada; e Department of Electrical and Computer Engineering, Western University, London, Ontario, Canada; f Cardiac Surgery Department, St Michael's Hospital, University of Toronto, Toronto, Ontario, Canada

**Keywords:** mitral valve, mitral valve repair, mitral valve surgery, mathematical model, simulation, training

## Abstract

**Objective:**

Effective mitral valve repair remains significantly operator-dependent partially due to the lack of standardized training substrates. We aimed to develop a simplified, parametric mathematical model of the mitral valve apparatus and physically validate its ability to accurately recreate the geometry and hemodynamic function of both a healthy valve and specified pathological valves when tested in a dynamic pulse duplicator system.

**Methods:**

A parametric mathematical framework was employed to define the 3-dimensional saddle-shaped annulus and its leaflet architecture using core, clinically measurable geometric coefficients (eg, annular diameters and segment-specific leaflet lengths). Five distinct silicone valve replicas were manufactured to match the mathematical specifications: 2 healthy baselines and 3 Carpentier Type II Prolapse valve variants (P1, P2, and P3) induced by localized leaflet length adjustments. Each physical model was tested in a dynamic pulse duplicator under physiological pressure and flow conditions. Valve function, geometry and regurgitation severity were quantified using cardiac ultrasound.

**Results:**

Derived measurements from the physical valve replicas accurately matched expected anatomical ranges from patient cohorts. The healthy baseline models consistently demonstrated competent function with no regurgitation. Pathological models, generated solely by manipulating the core geometric coefficients (lengthened P-segment, increased annular diameter), consistently exhibited characteristic segmental prolapse and moderate to severe mitral regurgitation. The resulting functional metrics, including regurgitant jet severity, confirmed the predictable functional outcome driven by the specified geometric inputs.

**Conclusions:**

We present a physically validated, anatomically configurable mathematical model that demonstrates a direct and predictable link between core geometric parameters and mitral valve functional behavior. Unlike existing physical simulation platforms that rely on biological tissue, this approach provides a standardized, reproducible, and customizable platform for surgical training and in vitro testing of novel repair techniques for segmental mitral valve disease.

## Graphical Abstract

**Figure:**
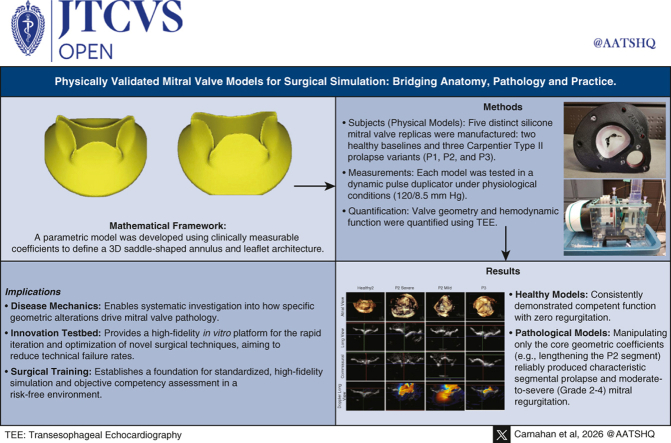

**Figure:**
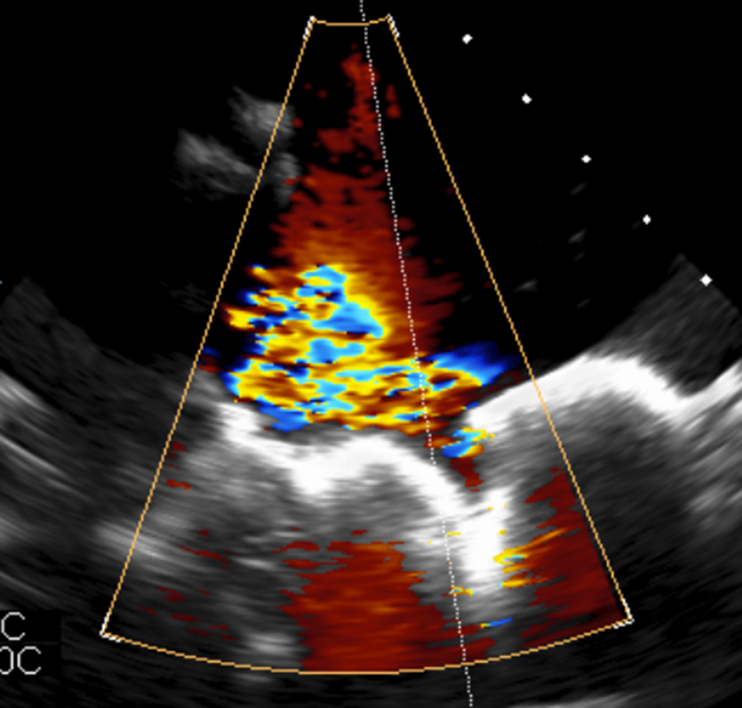
Validated P2 prolapse model showing characteristic regurgitant jet on ultrasound imaging.

**Central Message**

The core dimensions of a mitral valve are among the key factors affecting its function. Our validated model provides a reproducible substrate for surgical training and testing of repair strategies.

**Perspective**

Effective mitral valve repair remains operator-dependent. This study demonstrates that complex mitral valve functional behavior, including localized prolapse and regurgitation, can be accurately reproduced through core geometric parameters. The resulting validated model provides a customizable platform suitable for surgical skill mastery and in vitro optimization of repair techniques.

The mitral valve (MV) apparatus is a complex, dynamic functional unit where geometry dictates performance. Its proper function relies on the synchronized interplay of the annulus, leaflets, chordae, and papillary muscles within the changing geometry of the left ventricle.^1^ Pathological changes, such as leaflet prolapse or annular dilation, alter this delicate geometry, leading to hemodynamic failure like mitral regurgitation (MR). Understanding the precise geometric drivers of MR is crucial for successful surgical repair.

## The Clinical Problem and Simulation Need

Effective MV repair is a complex, operator-dependent procedure, particularly when addressing multiple segmental pathologies. Evidence indicates the volume of MV repair cases that a surgeon performs is a determinant not only of successful mitral repair rates, but also of freedom from reoperation and patient survival.^2^, ^3^, ^4^, ^5^ Current training for MV repair is limited to operating room observation; however, recent studies challenge this conventional approach and suggest introducing simulation as a way of ensuring resident's exposure to rare cases and high-risk procedures without compromising patient safety.^4^, ^5^, ^6^, ^7^, ^8^ Mastery of these advanced surgical techniques, especially those for complex Carpentier Type II prolapse (such as P1, P2, or P3 flail segments), is limited by the lack of realistic, standardized training models.^9^ Currently, the application of simulation technology in cardiac surgery can serve 2 primary purposes: computational modeling to study geometry-function relationships, and physical simulators for surgical training.^9^^,^^10^ However, both approaches struggle with similar limitations. Computational models, although valuable for understanding valve mechanics, often rely on oversimplified geometries. Similarly, existing physical simulators for training utilize low-fidelity models that lack anatomical realism or patient-specific data that is difficult to compile into standardized pathology libraries due to ethical constraints and data scarcity.^9^ A high-fidelity anatomical model paired with physical simulation could serve both roles: enabling systematic study of geometry-function relationships and providing a realistic substrate for surgical training.

## Current Modeling Limitations

Accurate mathematical models of the MV are highly valuable for studying the underlying physiology, anticipating pathologies, and creating physical replicas for training. Prior modeling efforts, including sophisticated finite element analysis or computational fluid dynamics, have been developed to enable the effects of therapy to be predicted in advance and to obtain a better understanding of MV physiology, with the goal of optimizing therapies. Several studies have been performed specifically modeling annuloplasty,^11^^,^^12^ edge-to-edge repair,^13^, ^14^, ^15^ and MitraClip (Abbott).^16^ Computational modeling studies have typically employed idealized geometries derived from mathematical models, or personalized geometries derived from limited clinical datasets.^1^^,^^17^^,^^18^ Physical simulation platforms for evaluating repair technique biomechanics have been demonstrated, notably including the 3-dimesionally (3D)-printed left heart simulator developed at Stanford,^19^^,^^20^ as well as our own left heart simulator as presented by Ginty and colleagues^21^ in 2018.^21^^,^^22^ However, these platforms rely on procured biological valves, live animals, or geometries derived from patient imaging. The absence of reproducible synthetic geometries such as those produced by mathematically defined valves leaves a gap for a standardized parametric substrate with controlled, replicable pathology. The overarching goal of research in this area has been to understand the significance of realistic material models and properties^23^, ^24^, ^25^, ^26^ as well as the geometry of the annulus and subvalvular apparatus as it relates to valve competence.^27^ Critically, many historical mathematical models of the MV fail to model both the leaflet geometry and the saddle-shaped annulus together in a single, unified design. Our group previously developed a parametric mathematical blueprint of the MV apparatus based on the work of Park and colleagues^18^ and Shen and colleagues,^28^ which unified the definition of the annular and leaflet geometry using core, clinically relevant coefficients.^29^ This built on earlier work demonstrating that patient-specific mitral valve geometries derived from clinical imaging could be physically replicated and tested in simulation, establishing that the pipeline from clinical measurement to physical model is feasible. The present study extends this foundation by validating that standardized, parametrically defined geometries, rather than patient-specific reconstructions, can reliably reproduce both healthy function and characteristic pathological behavior.

## Study Aim and Hypothesis

**Figure 1:**
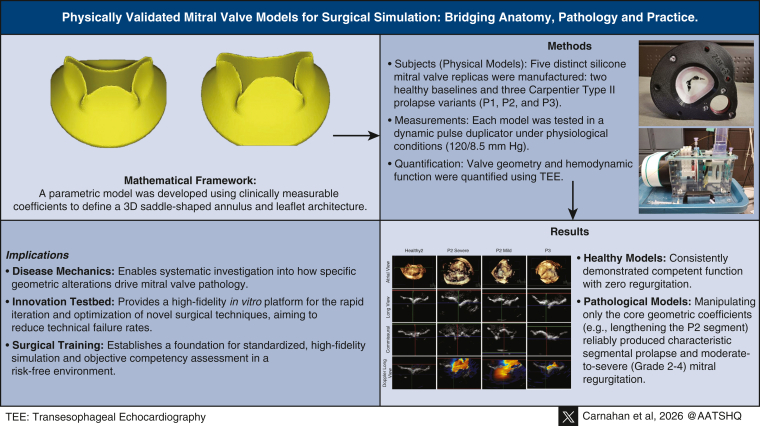
Graphical abstract showing, from top left: A computationally defined mitral valve profile, manufactured silicone replica of the valves and our left-heart simulator, and Doppler image showing regurgitation from valves tested in dynamic simulator.

This study builds on our mathematical framework by providing physical validation to bridge the gap between abstract modeling and practical surgical relevance. The aim is to develop a simplified, parametric mathematical model of the MV apparatus and physically validate its ability to accurately recreate the geometry and hemodynamic function of both a healthy valve and specified pathological valves (P1, P2, and P3 prolapse) when tested in a dynamic pulse duplicator simulator, demonstrated in Figure 1. We hypothesize that MV geometry and pathological functional behavior, as measured by standard clinical metrics, can be accurately dictated and predicted solely through the control of core geometric parameters.

## Methods

The research presented in this study did not involve human subjects, animal models, or the use of identifiable patient data. Accordingly, institutional review board approval was not required for this work. This study did not involve human participants or the use of clinical patient records; therefore, the requirement for informed written consent for the publication of study data was not applicable.

### Core Modeling Approach

**Figure E1:**
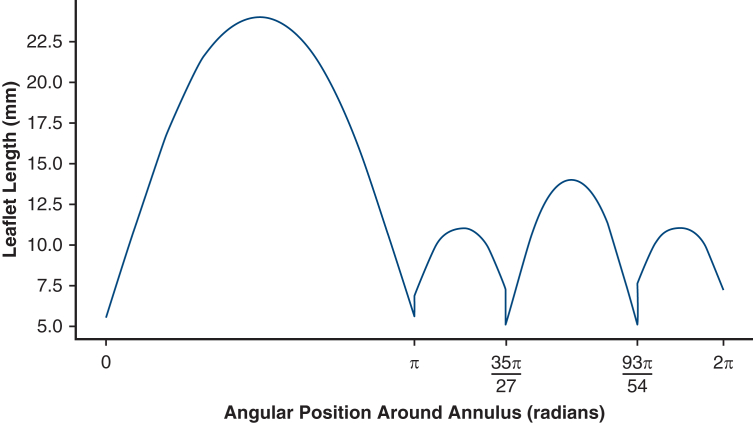
Flattened view of the leaflet geometries showing leaflet length as a function of angular position *u* (radians) around the mitral annulus The parameter *u* defines the angular position around the annular circumference, progressing from the anterior leaflet (*u* ∈ [0, π]) through the posterior segments P1, P2, and P3 (Carpentier Type II Prolapse valve variants) with segment boundaries marked at key angular values. For each angular position u, the corresponding leaflet length represents the scaled extent of the parameter *v*, which governs the normalized distance along the leaflet surface from annular base to free edge. Annular segmentations are defined at *u* ∈ [0, π] (anterior annulus), u ∈ [π, π × (35/27)] (anterolateral commissure to P1/P2 junction), *u* ∈ [π × (35/27), π × (93/54)] (central posterior segment), and *u* ∈ [π × (93/54), 2π] (P2/P3 junction to posteromedial commissure).

We developed a parametric mathematical model of mitral valve geometry that captures the valve's characteristic saddle-shaped annulus and billowing leaflets using 2 primary parameters: angular position around the annulus (*u*) and distance along each leaflet from base to free edge (*v*) (shown in Figure E1). This allows patient-specific customization while preserving anatomically realistic proportions.

### Annular Geometry

The mitral annulus has a characteristic 3D saddle shape, with anterior and posterior aspects sitting lower than the commissures, reducing leaflet stress during systole. We represented this geometry using a hyperbolic paraboloid surface anchored by 3 clinically measurable parameters: annular height (the elevation difference between commissures and mid-annulus), anterior-posterior (AP) diameter, and commissure-commissure (CC) diameter. This mathematical formulation (detailed in Appendix E1) ensures that the modeled annulus maintains the proper nonplanar contour observed in healthy valves, which is often flattened in disease states.

### Leaflet Architecture

We divided the valve into 4 anatomically distinct segments reflecting surgical nomenclature: the anterior mitral leaflet covering approximately one-third of the annular circumference, and 3 posterior segments (P1, P2, P3) each occupying roughly equal portions of the remaining two-thirds. This segmentation is clinically relevant because posterior leaflet pathology often localizes to specific segments (eg, P2 prolapse is the most common lesion requiring repair).

Each leaflet segment was generated using a paraboloid-based surface that naturally creates the curved, billowing shape observed in normal leaflets. Although all segments share the same underlying geometric structure, they are scaled differently to reflect anatomical proportions: the anterior mitral leaflet extends deeper into the ventricle (controlled by an anterior length coefficient), whereas the posterior segments are shorter but collectively cover more of the annular circumference (controlled by segment-specific length coefficients). For the commissural segments P1 and P3, we incorporated subtle length adjustments to account for the annular height changes at these transition zones, ensuring smooth leaflet contours without artificial discontinuities.

### Dynamic Coaptation Modeling

**Figure 2:**
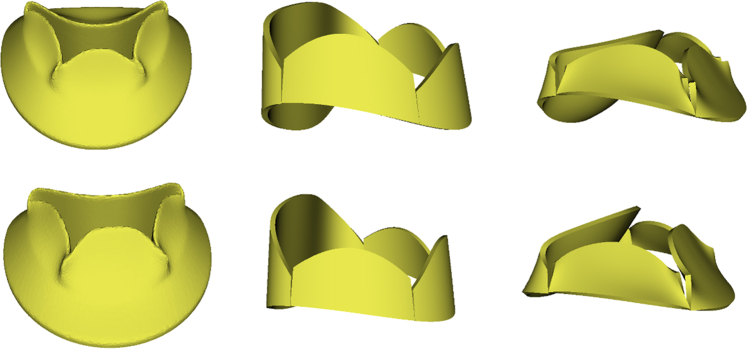
Mathematically defined valve geometries for the Healthy1 valve (transitional geometry with moderately enlarged annular parameters) (*top*) and Carpentier Type II Prolapse valve variant P2 (severe) (*bottom*). Left column shows combined 3-dimensional printable mold geometry, middle column shows fully open valve with vertical leaflets and 3-dimensional annular geometry, and column 3 shows leaflets at 75% closure. Key features of the P2 prolapse valve are the flattened and dilated annulus combined with elongated P2 leaflet.

Unlike static anatomical representations, this model simulates partial valve closure through adjustable closure parameters governing 3D leaflet displacement, achieving approximately 75% closure as demonstrated in Figure 2. Complete coaptation is intentionally excluded because this region requires computationally intensive finite element methods. Instead, our approach captures partial leaflet closure parametrically, with the final coaptation dynamics resolved through physical simulation. Closure incorporates both in-plane leaflet movement toward the central coaptation line and out-of-plane billowing under simulated ventricular pressure, detailed in Appendix E1.

### Clinical Parametrization

All model dimensions are defined by coefficients that correspond directly to measurements obtainable from standard echocardiographic or surgical assessments. This design philosophy ensures the model can be populated with patient-specific data or adjusted to represent various disease phenotypes (eg, dilated annulus in functional MR, excessive leaflet tissue in myxomatous disease). The complete mathematical formulations and equations for all surfaces are provided in Appendix E1.

### Pathological Model Generation (P1, P2, and P3 Prolapse)

To establish a functional library of pathologies relevant to surgical repair 5 distinct valve geometries were generated: 2 healthy valve baselines and 3 common Carpentier Type II Prolapse variants (P1, P2 Severe, and P2 Mild). The healthy valves were constructed using physiological anatomical dimensions to ensure sufficient coaptation length (typically 2-4 mm), with Healthy2 representing mean healthy dimensions and Healthy1 providing a transitional geometry with moderately enlarged annular parameters.

Prolapse was induced by manipulating segment-specific leaflet length coefficients. The length coefficient, which independently controls the length of the P1, P2, or P3 segments, was increased only in the targeted segment to match reported leaflet lengths in cases of severe regurgitation due to segmental prolapse. This localized increase in leaflet tissue simulates leaflet excess and dictates the mechanism of regurgitation by forcing the specific segment to extend past the coaptation line under simulated systolic pressure, accurately modeling the prolapse mechanism.

Annular parameters were progressively modified across the valve series to represent annular remodeling, a universal feature of pathological mitral valves. Multiple studies confirm that both the AP and CC diameters are significantly increased in patients with MR.^30^^,^^31^ In a comparative study of 112 subjects, patients with mitral valve prolapse and significant MR exhibited a mean AP diameter of 38.8 ± 6.4 mm and mean commissural width of 42.2 ± 5.9 mm, compared with 28.0 ± 2.5 mm and 33.3 ± 3.7 mm in healthy controls.^32^ Additionally, the normal saddle shape of the annulus becomes flattened in primary MV disease. This is quantified by the annular height to commissural width ratio (AHCWR), where lower values indicate greater flattening. Healthy controls exhibit a mean AHCWR of approximately 23.7%, whereas patients with significant MR show a reduced AHCWR of 13.2%.^32^ For all models, lengths for both the anterolateral and posteromedial papillary tips to the coaptation line as defined by Lee and colleagues^32^ were fixed at 20 mm. This value reflects reported physiological lengths and was held constant across all models deliberately, to isolate leaflet and annular geometry as the primary determinants of valve function. The influence of chordal length variation on model behavior remains an avenue for future investigation.

Our valve models reflect this progression from healthy to increasingly pathological states (Table 1). Healthy2 (AP, 28.0 mm; CC, 33.5 mm; AHCWR, 23.9%) closely matches healthy control dimensions, whereas the prolapse models demonstrate progressive annular enlargement and flattening: P2 Mild (AP, 33.0 mm; CC, 38.0 mm; AHCWR, 17.1%), P3 Prolapse (AP, 32.0 mm; CC, 41.0 mm; AHCWR, 14.6%), and P2 Severe (AP, 36.0 mm; CC, 42.0 mm; AHCWR, 13.1%). The P2 Severe model parameters align closely with the MR + cohort reported by Lee and colleagues,^32^ representing the most pathological geometry in our series. Segmental prolapse was superimposed on these progressively dilated annuli by selectively increasing leaflet lengths in the affected segments (Table 1).

**Table 1:** Selected parameters used to generate the 5 valve models for evaluation

Valve model	Annular dimension (mm)				Leaflet lengths (mm)			
	AP	CC	AH	AHCWR (%)	AML	P1	P2	P3
Healthy1	30.0	38.0	8.0	21.1	24.0	11.0	14.0	11.0
Healthy2	28.0	33.5	8.0	23.9	22.5	10.0	12.0	10.0
P3 Prolapse	32.0	41.0	6.0	14.6	25.0	12.0	16.5	17.0
P2 Severe	36.0	42.0	5.5	13.1	26.0	12.0	19.5	12.0
P2 Mild	33.0	38.0	6.5	17.1	25.0	11.5	17.0	11.5

*AP*, Anterior-posterior; *CC*, commissure-commissure; *AH*, annular height; *AHCWR*, annular height to commissural width ratio; *AML*, anterior mitral leaflet; *P1, P2, P3*, Carpentier Type II Prolapse valve variants; *Healthy1*, healthy baseline valve with transitional geometry with moderately enlarged annular parameters; *Healthy2*, healthy baseline valve with average dimensions.

### Pulse Duplicator Integration

**Figure 3:**
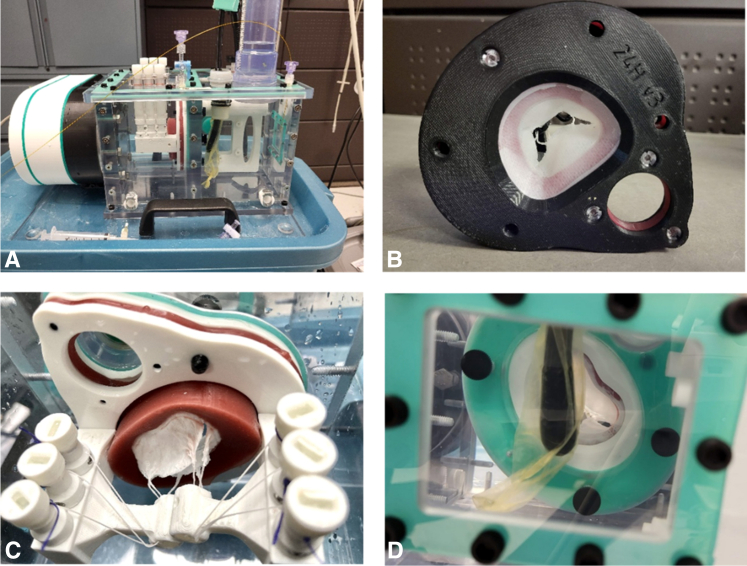
Silicone valve replica viewed in pulse-duplicator from above with chordae tensioners (A), and from atrial en face view (B). Pulse duplicator setup from side (C) and from en face view (D), including the transesophageal echocardiography probe used for evaluation.

A physical MV model (Archetype Biomedical Inc) is then manufactured to match the mathematical representations of each valve configuration. All valve configurations are generated at 75% closure for manufacturing purposes. Each valve geometry was manufactured as a single replica and measured postfabrication to validate geometric fidelity. The silicone valve replicas consist of molded silicone and gauze with 6 chordae tendineae, shown in Figure 3. After manufacturing the valve replica, we integrate it into a pulse-duplicator system that has been previously validated to produce a realistic pressure gradient across the mitral valve.^21^ The valve replica within the pulse duplicator is shown in Figure 3, as well as in Video 1. We then adjust chordae tendineae tensions for the 6 chordae integrated into the valve leaflets (2 anterior, 1 on each of P1 and P3, and 2 on the P2 leaflet) to best approximate healthy conditions with no regurgitation for the healthy condition valves, and to produce the expected characteristic regurgitation on the pathological valves. We use a transesophageal echocardiography probe to capture b-mode and color Doppler ultrasound to evaluate valve function in terms of regurgitation and derive valve measurements including annular area, annular circumference, tenting height, prolapse height, and prolapse volume (the 3D volume of leaflet tissue displaced above the annular plane during systole) using Philips QLab software (version 10.8.5, Philips Healthcare). Individual measurements are reported given the single-replica design. All echocardiographic images were reformatted to standardized multiplanar reconstruction axial views^33^ in Philips QLab version 10.8.5, ensuring orthogonal alignment and parallax elimination for consistent anatomical comparison. The pulse duplicator system is operated at ventricular pressures of 120 mm Hg and atrial pressures of 8.5 mm Hg measured using a healthy condition valve at 60 bpm, with a stroke volume of 60 mL. See Video 1 for a demonstration of the pulse duplicator system with the Healthy1 valve model in place. These conditions are kept constant for evaluation of the pathological valve models. Valve models are quantified via standard clinical measures, including annular circumference, annular area, tenting height, coaptation length, and billow height.

## Results

Selected parameters used as input for our mathematical models are reported in Table 1, with the resulting measured geometry of the physical silicone valve replicas reported in Table 2. Across all 5 valve replicas, derived measurements align with expected ranges based on reported valve geometry from patient cohorts, demonstrating the fidelity of our model in capturing valve morphology.

**Table 2:** Resulting measurements as obtained in the pulse duplicator on the silicone valve replicas using transesophageal echocardiography

Valve model	Annular area (mm^2^)	Annular circumference (mm)	Tenting height (mm)	Prolapse height (mm)	Prolapse volume (mL)	Regurgitation degree
Healthy1	860.6	122.8	8.1	2.4	0.2	0
Healthy2	766.3	104.1	3.9	3.4	0.6	0
P3 Prolapse	1147.4	135.6	7.6	4.4	1.3	2
P2 Severe	1274.1	137.9	4.7	12.9	3.6	4
P2 Mild	1175.0	134.0	2.7	5.3	1.9	3

*Healthy1*, Healthy baseline valve with transitional geometry with moderately enlarged annular parameters; *Healthy2*, healthy baseline valve with average dimensions; *P3, P2*, Carpentier Type II Prolapse valve variants.

The 2 healthy valve models (Healthy1 and Healthy2) demonstrated no regurgitation despite differences in annular dimensions. Healthy2, with an annular area of 766.3 mm^2^ and circumference of 104.1 mm, represented typical healthy valve dimensions, whereas Healthy1 showed moderately enlarged parameters (annular area, 860.6 mm^2^; circumference, 122.8 mm) yet maintained competency with minimal prolapse volume (0.2 mL). Both valves exhibited expected systolic and diastolic behavior with visually realistic appearance and dynamics.

**Figure 4:**
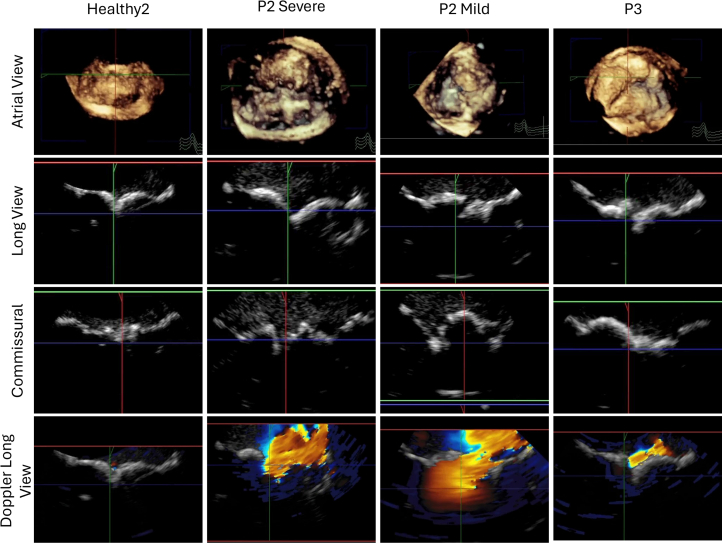
Standard axial images collected with transesophageal echocardiography probe of the Healthy2 valve (mean healthy dimensions) and each of the prolapse valve models (*columns*). Rows show axial view, long-axis, and commissural b-mode views, and a long-axis Doppler view for each valve.

The pathological valve replicas demonstrated characteristic billowing behavior with lack of coaptation along the lengthened posterior segments. The severity of regurgitation correlated with both the degree of annular dilatation and extent of segmental prolapse. P2 Mild (annular area, 1175.0 mm^2^; prolapse height, 5.3 mm; prolapse volume, 1.9 mL) showed moderate regurgitation (Grade 3), whereas P3 Prolapse (annular area, 1147.4 mm^2^; prolapse height, 4.4 mm; prolapse volume, 1.3 mL) exhibited moderate-to-severe regurgitation (Grade 2). P2 Severe, representing the most pathological geometry with the largest annular area (1274.1 mm^2^), greatest prolapse height (12.9 mm), and largest prolapse volume (3.6 mL), demonstrated severe regurgitation (Grade 4) as shown by b-mode imaging in Figure 4, *C* and color Doppler in Figure 4, *D*, with a maximum drop in systolic pressure to 83.0 mm Hg. The dynamic behavior and regurgitant jets under Doppler imaging are demonstrated in Videos 2-4.

## Discussion

We have developed a physically validated, anatomically configurable mathematical model that accurately reproduces healthy and pathological MV function through the control of core adjustable geometric parameters. Although numerous mathematical models of the mitral valve exist, including finite element models,^13^^,^^24^^,^^27^ analytical geometric model,^18^^,^^28^ and patient-specific reconstructions,^12^^,^^21^ direct comparison of their accuracy and realism remains challenging due to differences in modeling assumptions, validation approaches, and intended applications.^26^ Our parametric approach, combined with physical replication and testing in a pulse duplicator system, is rapid, reproducible, and provides a direct, causal link between anatomy and function that distinguishes it from purely computational approaches.

Using our mathematical valve models, we generated 5 valve geometries spanning the range of reported dimensions in healthy individuals and common pathological variants. Our 2 healthy valve models (Healthy1: annular area, 860.6 mm^2^; AHCWR, 21.1%; and Healthy2: annular area, 766.3 mm^2^; AHCWR, 23.9%) demonstrated no regurgitation despite differences in annular dimensions, validating that both geometries fall within the functional range. Derived measurements from these replicas matched expected clinical values, with AHCWR closely approximating the 23.7% reported in healthy cohorts.^31^ In our pathological valve series, we demonstrated that adjusting leaflet lengths and annular dimensions to approximate segmental leaflet prolapse with progressive annular remodeling creates characteristic regurgitant behavior. The P2 Mild model (prolapse height, 5.3 mm; prolapse volume, 1.9 mL) exhibited less-severe regurgitation (Grade 2), whereas P2 Severe (prolapse height, 12.9 mm; prolapse volume, 3.6 mL; AHCWR, 13.1%) demonstrated severe regurgitation (Grade 4) with annular parameters matching the MR + cohort reported by Lee and colleagues.^32^ This progression demonstrates both the realism and functionality of the mathematically defined valve geometries and provides proof of concept for exploring geometry-function relationships in the mitral valve.

### Clinical Significance

Traditional training methods rely on animal models or cadaveric tissue, which have high cost, ethical constraints, lack of standardization across training sessions, and lack of controlled pathologies resembling realistic clinical scenarios.^4^^,^^8^ Several in vitro mitral valve simulators have been developed,^21^^,^^34^, ^35^, ^36^ but these typically use fixed anatomies or rely on procured biological tissue with uncontrolled pathology. In contrast, our model provides a platform where anatomical and mechanical properties can be tuned and consistently replicated across severity grades, as demonstrated by our ability to generate both mild and severe P2 prolapse variants with quantifiable differences in prolapse geometry. This capability is valuable for developing proficiency in complex repair techniques for segmental diseases, allowing trainees to practice repeatedly on identical pathologies with progressive difficulty. The current chordal architecture also permits simulation of anterior leaflet pathology; A1, A2, or A3 prolapse could be induced by selectively lengthening marginal chords to those segments, extending the platform's utility to repairs that are typically more technically demanding than isolated posterior disease. Similarly, Type IIIB dysfunction from secondary disease could be approximated by tightening the marginal and secondary chords, enabling simulation of restricted leaflet motion. Bileaflet and anterior Type II prolapse represent natural extensions of the current model that warrant systematic investigation. Expanding the pathology library in these directions would meaningfully broaden the range of repair scenarios available for structured, repeatable training.^4^^,^^5^

A significant potential utility lies in the model's application as a testing platform for optimizing repair techniques in silico (through computational analysis) or in vitro (on the physical model) before clinical application. In silico platforms for surgical planning have shown promise in cardiac interventions,^17^ but validation against physical models remains limited. Technical failure in complex surgical reconstructions is often multifactorial but can be significantly influenced by suboptimal technique or material choice.^2^^,^^3^^,^^30^ Our platform bridges this gap by enabling systematic variation of parameters such as suture placement, material stiffness, or device geometry with immediate observation of biomechanical consequences. For instance, the P3 Prolapse model (prolapse height, 4.4 mm; Grade 3 regurgitation) provides a controlled substrate for testing P3 repair techniques, whereas the range from P2 Mild to P2 Severe enables assessment of repair efficacy across disease severity. This systematic preoperative testing can identify the most robust and physiologically sound repair strategy for a given pathology, offering a pathway to reduce technical failure rates and enhance long-term patient outcomes.

A key finding is that regurgitation severity followed predictably from geometric inputs alone. Previous studies have correlated specific anatomical measurements with valve dysfunction, but direct manipulation of these parameters in validated physical models has been limited. Our demonstration that progressive annular dilatation combined with segmental leaflet elongation produces predictable gradations of regurgitant severity validates a causal rather than merely correlative relationship that provides a rational basis for repair strategies targeting specific geometric abnormalities.^31^^,^^32^

### Limitations and Future Work

This study has several limitations that must be considered when interpreting results and translating findings to clinical applications. Only 1 physical replica of each valve geometry was manufactured and tested, precluding statistical comparison of measured parameters with patient cohort data through formal hypothesis testing and preventing quantification of manufacturing variability. However, the measured geometric values fall within clinically reported ranges for their respective pathological categories, supporting the fidelity of our manufacturing approach. Future work incorporating multiple replicas per geometry would enable more robust statistical validation and assessment of manufacturing reproducibility.

The model employs fixed chordal length and stiffness, which enables repeatable experiments and simplifies fabrication but fails to replicate the complex biomechanical reality of native chordae tendineae. Native chordae exhibit nonlinear, anisotropic, and viscoelastic behavior that dynamically responds to load. This lack of dynamic material fidelity limits the model's capacity to predict nuanced structural failure modes or accurately simulate long-term remodeling and creep often observed in diseased or repaired biological tissue. Future iterations incorporating force transducers on the chordae would allow quantification of tensile loads during closure, providing a more complete biomechanical characterization of repair strategies. A critical physiological abstraction is the absence of comprehensive left ventricular dynamics and myocardial contractility within the pulse duplicator system. Replacing the contracting ventricle with fixed pressure-flow conditions decouples repair mechanics from cardiovascular feedback. Although the model can rigorously test structural durability under predefined loads, it cannot simulate how an optimized repair might improve cardiac efficiency or how a suboptimal repair might lead to increased ventricular afterload.

Finally, the selection of silicone as the replica material introduces inherent material property discrepancies. Despite silicone's excellent tunability and mechanical consistency across fabrication batches, it is an isotropic, linear elastic polymer fundamentally distinct from the heterogeneous, nonlinear, and actively remodeling collagenous and muscular tissue of the native heart. This distinction is particularly relevant for the prolapse models presented here: myxomatous leaflets are not simply elongated but exhibit fundamentally altered collagen architecture and increased mucin deposition that substantially change their mechanical behavior under load. Representing prolapse through geometric elongation of a homogeneous silicone structure therefore captures the anatomical consequences of myxomatous disease without replicating its biomechanical substrate. This material mismatch means the model cannot fully reproduce complex anisotropic stress-strain relationships, rate-dependent viscoelasticity, or long-term biological processes such as calcification, degradation, or cellular response. Therefore, whereas the silicone replica is ideal for surgical training and gross mechanical testing, results must be extrapolated cautiously when predicting chronic in vivo durability and biological integration of surgical repairs or implanted devices.

Several important extensions of this work remain to be demonstrated. Most immediately, the pathological models described here provide a controlled substrate on which repair strategies can be executed and evaluated. Demonstrating that regurgitation can be reliably eliminated through standard repair techniques, such as those we have previously validated on patient-specific physical valve models, would substantially strengthen the case for this platform as a surgical training tool and represents a clear next step. Additionally, formal studies evaluating educational efficacy would be necessary to validate the value of these models in surgical training. The standardized parametric approach taken here is also a deliberate step toward broader accessibility. Unlike patient-specific reconstruction, which requires individual clinical datasets, the parametric framework allows any clinician to generate anatomically realistic models from routine echocardiographic measurements. The parametric coefficients correspond to clinically obtainable dimensions, meaning the parametric framework can be populated with patient-specific data to enable individualized surgical rehearsal. This is an alternative to the direct patient-specific modelling via segmentation of the valve from diagnostic transesophageal echocardiography that our group has previously explored,^22^^,^^37^ and represents a natural extension of this work.

## Conclusions

The validated parametric model establishes that the functional behavior of this segmental disease is a direct, predictable consequence of its core geometric dimensions. By confirming this geometric-functional relationship, this work provides a reproducible, standardized parametric valve model that advances beyond descriptive analysis to enable systematic, controlled investigation of how core geometric parameters drive MV dysfunction. This platform has 3 main applications: investigating disease mechanics, serving as an in vitro testbed for rapid iteration and optimization of novel surgical techniques to reduce technical failure, and providing a foundation for high-fidelity simulation for surgical training and competency assessment. Together, these applications position the model as a practical tool for both the surgical training environment and the research laboratory.

### Declaration of Generative AI and AI-Assisted Technologies in the Writing Process

During the preparation of this work the author(s) used large language models, including Google Gemini 2.5 Flash and Claude Sonnet 4.5 to edit, polish, and refine the manuscript draft, including abstract and discussion sections, for clarity, conciseness, and tone consistency. After using this tool/service, the authors reviewed and edited the content as needed and take full responsibility for the content of the publication.

### Webcast

You can watch a Webcast of this AATS meeting presentation by going to: https://www.aats.org/resources/physically-validated-mitral-va-11357.

**Figure:**
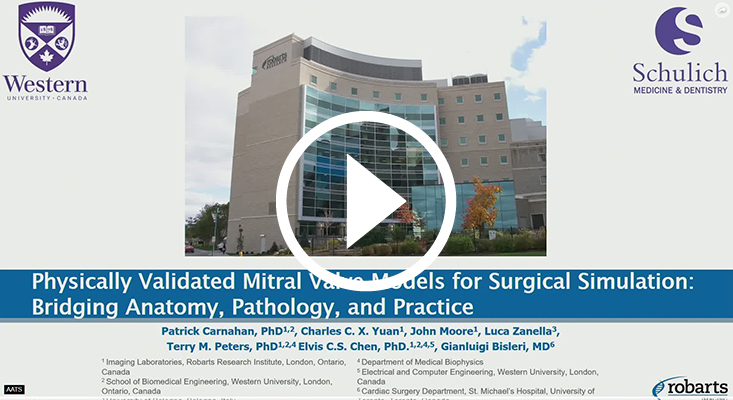

## Conflict of Interest Statement

Mr Moore and Drs Chen, Peters, and Bisleri are co-owners of Archetype Biomedical Inc. All other authors reported no conflicts of interest.

The *Journal* style requires editors and reviewers to disclose conflicts of interest and to decline handling or reviewing manuscripts for which they may have a conflict of interest. The editors and reviewers of this article have no conflicts of interest.

## Controllable Parameters

The mathematical model allows control over several key geometric parameters that define the mitral valve's anatomy and function. These include annular dimensions (commissure-commissure diameter [CCdiam], anteroposterior diameter [APdiam], and annular height [AH]), leaflet lengths (anterior leaflet length [ALlength], P1 length, P2 length, and P3 length), curvature coefficients, and dynamic closure adjustments (Δx, Δy, and Δz) and coaptation factor. The model enables independent manipulation of each parameter, facilitating the generation of a wide range of healthy and pathological valve geometries.

### Annular Geometry

Annular saddle shape is described by:

$$Ann\left(x,y\right)=\frac{{\left(x-al\right)}^{2}}{\left(annulus_{a}\cdot av\right)}-\frac{{\left(y-bl\right)}^{2}}{\left(annulus_{b}\cdot bv\right)}$$

where annulus_a_ = (CCdiam/2)^2^/AH, annulus_b_ = (APdiam/2)^2^/AH, al = 0, bl = -APdiam × 1.25, *av* = 1 and *bv* = APdiam × 0.75. This equation generates the characteristic saddle shape of the mitral annulus, with the *u* parameter defining the angular position around the annulus.

## Closure Parameter Description

Valve closure is simulated by dynamically scaling and shifting the leaflet coordinates using a set of parametric adjustments. The closure process is controlled by a closure parameter ii (ranging from 0 to 24, where 0 is fully open and 24 is approximately 75% closed) and the leaflet's position relative to the annular plane (defined by *x* and *y*). The leaflet height is reduced as closure progresses, approximated by an ellipsoidal fit. The term xr=(1/0.2)·cos(θ) and yr=0.8·(1/0.2)·sin(θ), θ∈[0,2π] are used to scale the leaflet's extent in the *x* and *y* directions. This ensures the leaflet height decreases smoothly as the valve closes. Because the closure simulation includes slight billowing, these terms ensure that the overall leaflet length, including curvature, remain constant.

### Leaflet Structure

All leaflet surfaces are generated using a paraboloid-based structure, which is then scaled and adjusted to reflect anatomical size and enable dynamic coaptation. The leaflet geometry is controlled by the *v* parameter, whose range is limited depending on the value of *u*, allowing the hyperbolic paraboloid to be cut and shaped into realistic leaflet segments.

### Anterior Mitral Leaflet

The anterior mitral leaflet (AML), which covers approximately one-third of the anteroposterior diameter, is defined by the following equations:

$$x=\left(\frac{CCdiam}{2}\right)\sqrt{v}\cos\left(u\right)+\Delta x_{AML}$$

$$y=\left(APdiam+AC\right)\cdot 0.33\cdot \sqrt{v}\sin\left(u\right)-AC\cdot \exp\left(-\frac{{\left(u-\pi /2\right)}^{2}}{2\cdot 0.5^{2}}\right)-\Delta y_{AML}$$

$$z=\left(-ALlength\cdot \;xr\left(i\right)\;\cdot \;v+ALlength\;\cdot \;xr\left(i\right)\right)+Ann\left(x,y\right)-{\Delta z}_{AML}$$

$${\Delta x}_{AML}=\left(0.3\cdot i\right)\cdot \left(1-v\right)\cdot \text{sign}\left(\cos\left(u\right)\right)\cdot \left(0.9-{\left|\frac{\pi /2-u}{\pi /2}\right|}^{2}\right)$$

$$\Delta y_{AML}=ALlength\cdot 1.1\cdot yr\left(i\right)\cdot \left(1-v\right)\cdot \left(0.9-{\left|\frac{\pi /2-u}{\pi /2}\right|}^{2}\right)$$

$$\Delta z_{AML}=\sin\left(u\right)\cdot \sin\left(\left(v-\text{ratio}\right)\cdot \frac{\pi }{0.2}\right)\cdot 4\cdot i\cdot \frac{\pi /2}{48}\cdot \left(0.9-{\left|\frac{\pi /2-u}{\pi /2}\right|}^{2}\right)$$

where u∈[0,π], v $\in \left[1-\frac{\left(\sin\left(u\right)+0.3\right)}{1.3},1\right]$.

### Posterior Mitral Leaflets P1, P2, P3

The posterior mitral Leaflet (PML) segments, which cover approximately two-thirds of the APdiam, are defined by modifying the AP scaling factor:

$$x=\left(CCdiam/2\right)\sqrt{v}\cos\left(u\right)+\Delta x_{PML}$$

$$y=\left(APdiam+AC\right)\cdot 0.66\cdot \sqrt{v}\sin\left(u\right)-\Delta y_{PML}$$

$$z=\left(-PL_{length}\cdot xr\left(i\right)\cdot v+PL_{length}\cdot xr\left(i\right)\right)+Ann\left(x,y\right)-\Delta z_{PML}$$

$${\Delta x}_{PML}=\left(0.2\cdot i\right)\cdot \left(1-v\right)\cdot sign\left(\cos\left(u\right)\right)\cdot \left(1-{\left|\frac{\left(\pi /2-u\right)}{\left(\pi /2-\pi \cdot \left(35/27\right)\right)}\right|}^{\left(4\cdot CF\right)}\right)$$

$$\Delta y_{PML}=\sin\left(u\right)\cdot PL_{length}\cdot 1.1\cdot yr\left[i\right]\cdot \left(1-v\right)\cdot \left(1-{\left|\frac{\left(\pi /2-u\right)}{\left(\pi /2-\pi \cdot \left(35/27\right)\right)}\right|}^{\left(4\cdot CF\right)}\right)$$

$$\Delta z_{PML}=\sin\left(u\right)\cdot \sin\left(\left(v-ratio\right)\cdot \left(\frac{\pi }{0.2}\right)\right)\cdot 3.5\cdot i\cdot \left(\frac{\left(\pi /2\right)}{48}\right)\cdot \left(0.5-{\left|\frac{\left(\pi /2-u\right)}{\left(\pi /2-\pi \cdot \left(35/27\right)\right)}\right|}^{\left(4\cdot CF\right)}\right)$$

Where u ∈ [π, π × (35/27)], v∈[1−0.2·sin(1.9·(u−π+0.35)),1] (P1 segment), u ∈ [π × (35/27), π × (93/54)], $v\in \left[1-0.2\cdot \left(\frac{15}{14}\cdot \sin\left(\frac{\pi }{9}\right)+\left(1-\frac{15}{14}\cdot \sin\left(\frac{\pi }{9}\right)\right)\cdot \sin\left(\frac{54}{23}\cdot \left(u-\frac{35}{27}\cdot \pi \right)\right)\right),1\right]$ (P2 segment) and u ∈ [π × (93/54), 2π], v∈[1−0.2·sin(1.9·(π+0.35−u)),1] (P3 segment) for the P1, P2 and P3 leaflets respectively

Collectively, these parameters and equations define the geometry of the mitral leaflets in 3-dimensional space. After generating the geometry, it is converted into a faceted mesh format using the visualization toolkit, representing the medial surface of the leaflets. The inner mold is then generated to fill the atrial side of the valve geometry, enabling 3-dimensional printing and silicone valve manufacturing.

## Supplementary Data

Video 1 Demonstration of pulse duplicator system with Healthy1 valve model (transitional geometry with moderately enlarged annular parameters) and transesophageal echocardiography probe in place. Video available at: https://www.jtcvs.org/article/S2666-2736(26)00169-5/fulltext.

Video 2 Doppler ultrasound showing regurgitant jet for the Carpentier Type II Prolapse valve variant P2 (severe) valve demonstrating characteristic jet formation with severe regurgitation. Video available at: https://www.jtcvs.org/article/S2666-2736(26)00169-5/fulltext.

Video 3 Doppler ultrasound showing regurgitant jet for the Carpentier Type II Prolapse valve variant P2 (mild) valve demonstrating reduced severity regurgitation. Video available at: https://www.jtcvs.org/article/S2666-2736(26)00169-5/fulltext.

Video 4 Doppler ultrasound showing regurgitant jet for the Carpentier Type II Prolapse valve variant P3 valve demonstrating moderate regurgitation. Video available at: https://www.jtcvs.org/article/S2666-2736(26)00169-5/fulltext.
