# Supplementary figures and images for: Physically validated mitral valve models for surgical simulation: Bridging anatomy, pathology, and practice
Source: JTCVS Open. 2026 Apr 1;31:101746. doi: 10.1016/j.xjon.2026.101746 (PMC13316320; doi:10.1016/j.xjon.2026.101746)

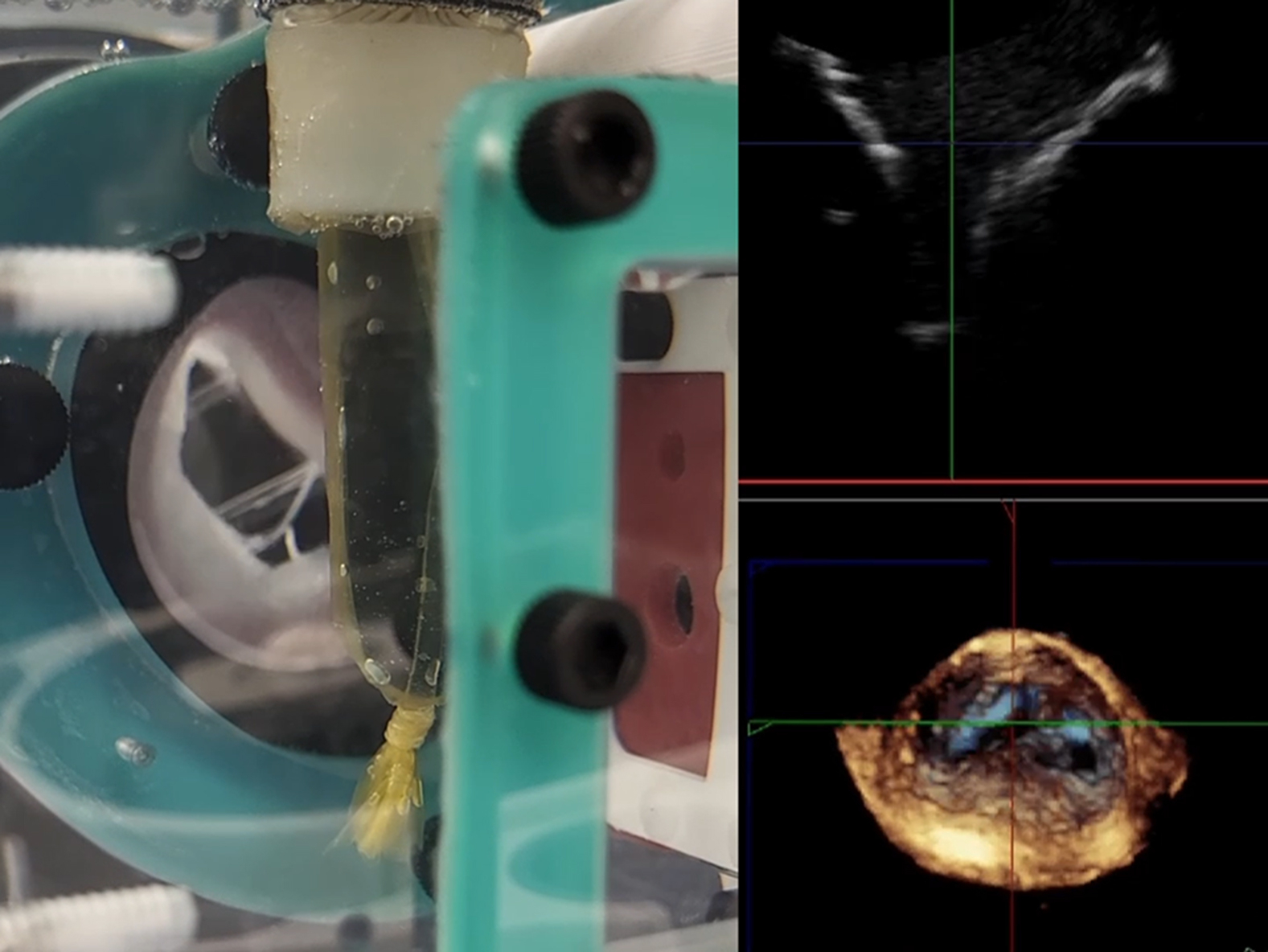

Supplement: Video 1 — Demonstration of pulse duplicator system with Healthy1 valve model (transitional geometry with moderately enlarged annular parameters) and transesophageal echocardiography probe in place. Video available at: https://www.jtcvs.org/article/S2666-2736(26)00169-5/fulltext. [file fx3.jpg]

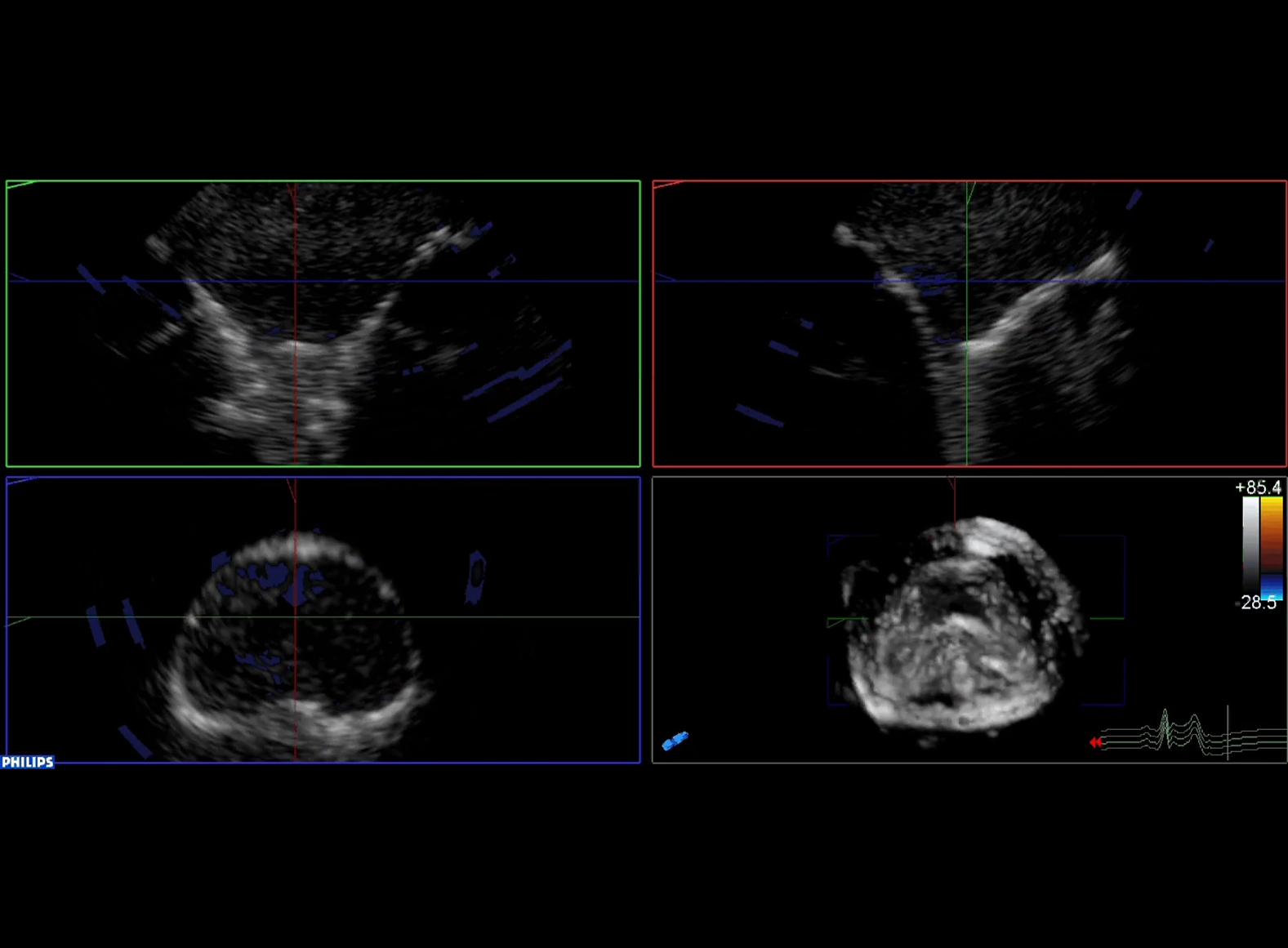

Supplement: Video 2 — Doppler ultrasound showing regurgitant jet for the Carpentier Type II Prolapse valve variant P2 (severe) valve demonstrating characteristic jet formation with severe regurgitation. Video available at: https://www.jtcvs.org/article/S2666-2736(26)00169-5/fulltext. [file fx4.jpg]

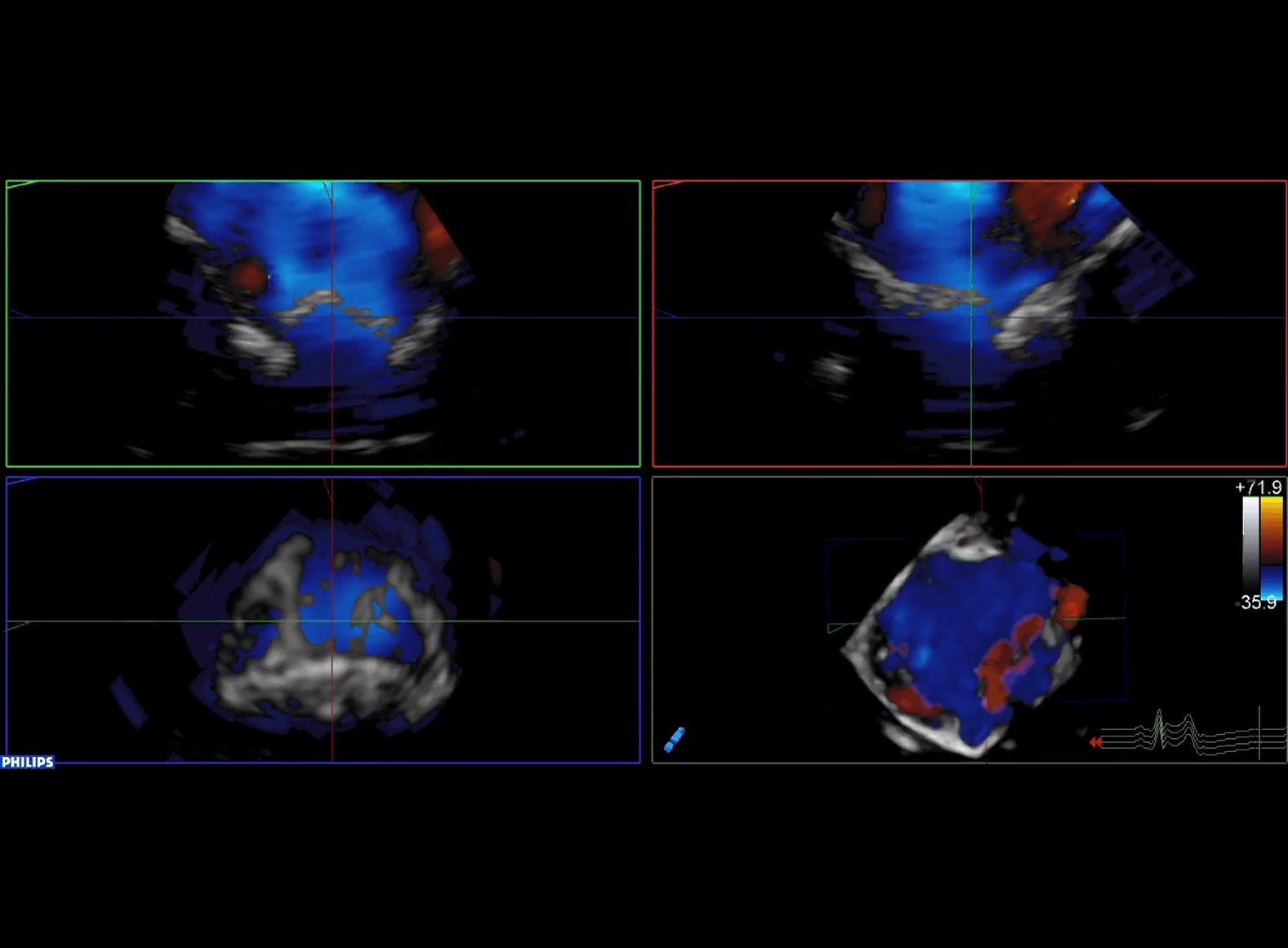

Supplement: Video 3 — Doppler ultrasound showing regurgitant jet for the Carpentier Type II Prolapse valve variant P2 (mild) valve demonstrating reduced severity regurgitation. Video available at: https://www.jtcvs.org/article/S2666-2736(26)00169-5/fulltext. [file fx5.jpg]

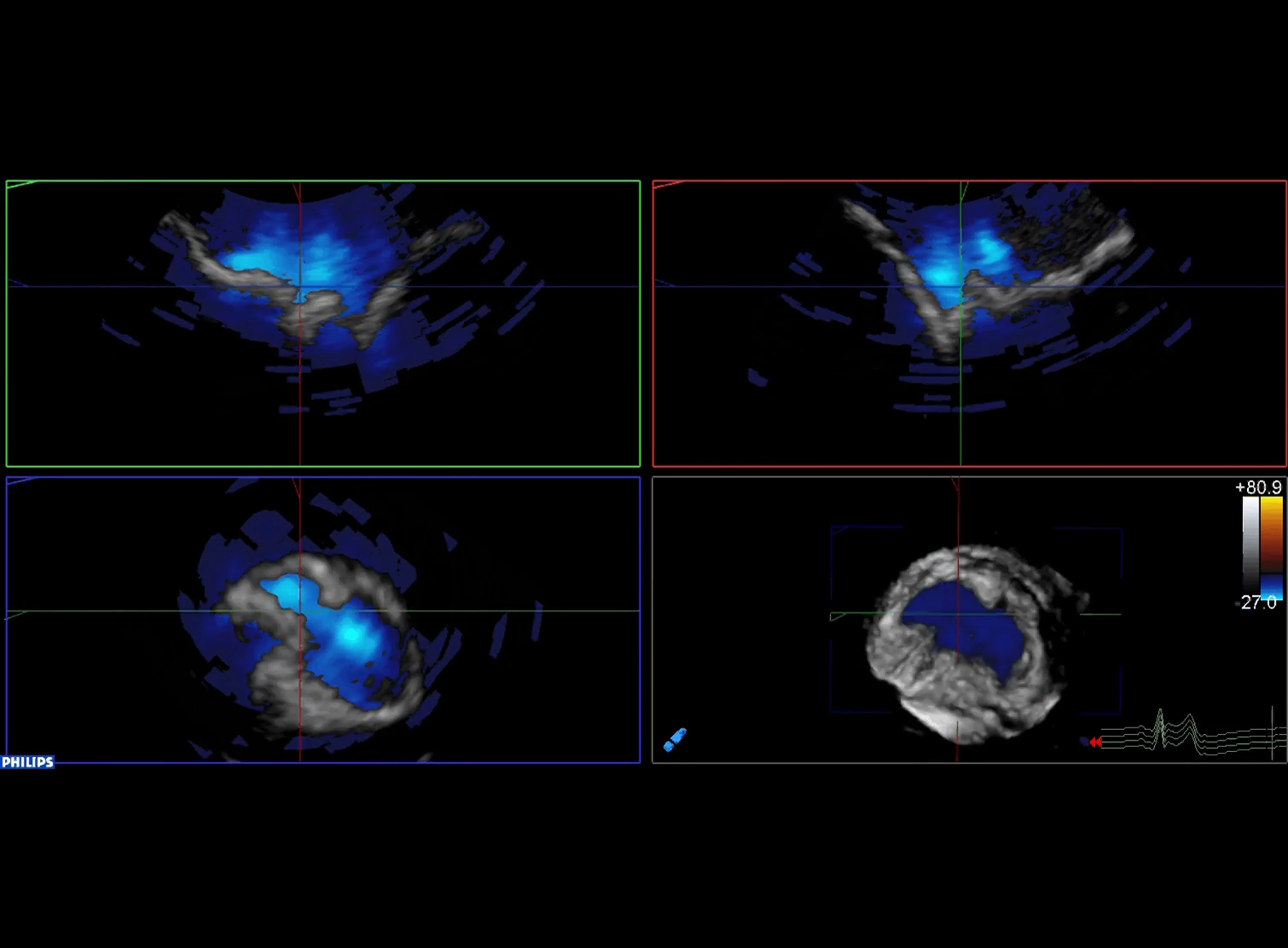

Supplement: Video 4 — Doppler ultrasound showing regurgitant jet for the Carpentier Type II Prolapse valve variant P3 valve demonstrating moderate regurgitation. Video available at: https://www.jtcvs.org/article/S2666-2736(26)00169-5/fulltext. [file fx6.jpg]
